# Abnormalities of white matter microstructure in unmedicated patients with obsessive–compulsive disorder: Changes after cognitive behavioral therapy

**DOI:** 10.1002/brb3.1201

**Published:** 2019-01-09

**Authors:** ZhaoXi Zhong, XiangYun Yang, RuiXiang Cao, Ping Li, ZhanJiang Li, LuXian Lv, Dai Zhang

**Affiliations:** ^1^ Psychiatry Institute of Mental Health/Peking University Sixth Hospital, Peking University Beijing China; ^2^ Henan Key Lab of Biological Psychiatry The Second Affiliated Hospital of Xinxiang Medical University Xinxiang China; ^3^ The China Clinical Research Center for Mental Disorders & Beijing Key Laboratory of Mental Disorders Beijing Anding Hospital, Capital Medical University Beijing China; ^4^ Department of Psychiatry Qiqihar Medical University Qiqihar China

**Keywords:** cognitive behavioral therapy, diffusion tensor imaging, obsessive–compulsive disorder, white matter

## Abstract

**Background:**

Cognitive behavioral therapy (CBT) is an effective treatment for Obsessive–compulsive disorder (OCD). Structural and functional white matter defects may suggest a vital neurobiological basis of OCD. However, the effects of CBT on white matter in OCD remain unknown.

**Objective:**

The aim was to investigate white matter changes and the effect of CBT on white matter in OCD patients.

**Methods:**

Fractional anisotropy (FA) maps were acquired using DTI. Participants included 85 patients with OCD and 90 healthy controls. VBM was then performed to detect regions with significant group differences.

**Results:**

Obsessive–compulsive disorder patients exhibited significantly reduced FA values in bilateral OFC, right cerebellum, and left SPG, while higher FA values were observed in right PUT compared with healthy controls. Following CBT, OCD patients showed higher FA values in right MFG, left OFC, right cerebellum, and left MTG, and decreased FA values in right PUT in comparison with pretreatment. Furthermore, FA values in the left OFC of patients were significantly positively correlated with the Y‐BOCS and its associated Compulsions subscale, and FA values in the right PUT were positively correlated with Compulsions subscale. In addition, the percentage change in FA values in left MTG was positively correlated with the percentage reduction in Compulsions subscale, while the percentage change in FA values in left OFC and right PUT was negatively correlated with the percentage reductions in Obsessive and Compulsions subscale, respectively.

**Conclusions:**

Our findings demonstrate the abnormalities of white matter microstructure in unmedicated patients with OCD. These abnormalities may be partly reversed by CBT.

## 1 INTRODUCTION

Obsessive–compulsive disorder (OCD) is a chronic psychiatric disorder and associated with significant anxiety, distress, and social dysfunction (Weissman, Bland, Canino, & Greenwald, 1994). The worldwide prevalence is 2%–3% (Horwath & Weissman, 2000). However, the neurobiological mechanisms of OCD are still unknown. Several studies revealed a positive correlation between white matter (WM) alterations and the severity of OCD symptoms. In these studies, Garber et al found the differences in the orbital PFC were strongly correlated with symptom severity in OCD patients (Garber, Ananth, Chiu, & Griswold, 1989). Duran et al found that patients’ symptom was positively correlated with WM volume in the anterior limb of the internal capsule (ALIC; Duran, Hoexter, Valente, Miguel, & Busatto, 2009). MRS studies found that OCD patients showed WM abnormalities in frontal (Whiteside, Port, Deacon, & Abramowitz, 2006) and parietal (Kitamura et al., 2006), which were positively correlated with the severity of symptoms. All aforementioned studies suggest that the defects of WM may suggest a neurobiological basis for OCD.

Diffusion tensor imaging (DTI) is a noninvasive technique to investigate WM microstructures. DTI is sensitive to the orientation and integrity of the underlying WM fiber in vivo. Fractional anisotropy (FA) is the most commonly used index to detect this anisotropy and is deemed a sensitive marker of changes in tissue microstructures. Some DTI studies exhibited WM integrity abnormalities in some brain regions within the fronto‐striato‐thalamo‐cortical loop and other regions (e.g., limbic, parietal, cerebellar) in OCD patients. For example, Szeszko et al. (2005) identified that OCD patients showed lower FA bilaterally in the ACC, parietal gyrus, right PCG, and left lingual gyrus. Cannistraro et al. (2007) found significantly greater FA in the cingulate bundle bilaterally and in the left anterior limb of the internal capsule of patients. Lochner et al. (2012) found that OCD patients exhibited increased FA in bilateral ALIC as well as decreased FA in the right anterior limb near the head of the caudate. Saito et al. (2008) observed a significant reduction in FA in the rostrum of the corpus callosum of patients. Garibotto et al. (2010) revealed that patients showed significantly lower FA in the corpus callosum (CC), cingulum, superior longitudinal fasciculus, and inferior fronto‐occipital fasciculus bilaterally. Fan et al. (2012) found that OCD patients exhibited decreased FA in left SFG, temporo‐parietal lobe, and MOG as well as striatum WM. Using tract‐based analyses, Benedetti et al. (2013) found that drug‐treated patients showed widespread FA decreases and RD increases compared to drug‐naïve patients and healthy controls. Fontenelle et al. (2011) found that patients showed decreased FA and increased MD in genu of capsula interna. In some studies, patients showed decreased FA in body of CC (Bora et al., 2011), increased FA in genu and body of CC (Li et al., 2011), and decreased FA in anterior body of CC (Nakamae et al., 2011), respectively. In another studies, patients showed increased AD in CC (Jayarajan et al., 2012) and decreased AD in CC (genu, splenium; Silk, Seal, & Vance, 2013) respectively. In meta‐analyses, Radua et al. (2007) reported that patients with OCD may have widespread WM abnormalities, which are particularly prominent in anterior midline structures. The findings are broadly consistent with both the classic fronto‐striatal model of the disorder and also with more recent “systems” approaches, which emphasize the implication of multiple other brain systems (fronto‐limbic, fronto‐parietal) in OCD. These studies outline a mechanism for the WM disconnections found in the brains of OCD patients.

Short range and maneuverable CBT has been recognized as an effective treatment for OCD (Bolton & Perrin, 2008). Based on cognitive theory, this treatment adopts cognitive and behavioral methods and techniques to address cognitive disruption, achieving a recovery rate of 58%–76% (Whittal, Thordarson, & McLean, 2005). Despite these benefits, a biological perspective of the mechanisms of CBT in OCD patients has yet to be defined.

Several neuroimaging studies have indicated that CBT could induce anatomical and functional changes in the brains of OCD patients. Hoexter et al. (2012) found that abnormalities in gray matter volume in the left putamen were no longer detectable after CBT. Functional MRI (fMRI) studies have elaborated upon findings that outline the effects of CBT on brain activity in OCD patients. Freyer et al. (2011) found that the caudate nucleus showed increased activity in OCD patients after CBT. In addition, Olatunji et al. (2014) found that activation in the anterior temporal pole and amygdala was most strongly associated with a better treatment response to CBT. A further fMRI study found that the hemodynamic response of the left OFC and ACC to obsession‐inducing images decreased following a three‐month course of CBT (Morgieve, Haynes, Granger, Clair, & Pelissolo, 2014). According to the results outlined above, an effective CBT program generates changes in the structure and function of these brain regions, thereby leading to an improvement in symptoms. However, no study has examined WM changes in these brain regions or tissues following treatment with CBT. As these brain regions are connected by WM fibers, we hypothesized that an effective course of CBT would restore the connection of WM fibers in the fronto‐striato‐thalamo‐cortical circuit and other regions (e.g., limbic, parietal, cerebellar) of OCD patients and facilitate the recovery of function and structure, thus leading to an improvement in symptoms.

We utilized the DTI method to investigate WM abnormalities located within the fronto‐striato‐thalamo‐cortical circuit of patients with OCD both before and after CBT.

## 2 METHODS

### 2.1 Participants

The study included 85 OCD patients (female/male: 33/52; mean age: 27.6 ± 6.7 years) and 90 healthy controls (female/male: 25/65; mean age: 28.2 ± 6.8). The Structured Clinical Interview from the DSM‐IV Axis I Disorders was carried out and all patients satisfied the DSM‐IV diagnostic criteria for OCD (DSM‐IV 1994). All participants attended the outpatient clinic at Capital Medical University, Beijing Anding Hospital and the second affiliated hospital of Xinxiang Medical University from August 2009 until May 2017. The Yale‐Brown Obsessive Compulsive Scale (Y‐BOCS) (Goodman et al., 1989) was used to assess symptom severity. Only patients who scored ≥16 on the Y‐BOCS were included. Each patient completed the 17‐item Hamilton Depression Rating Scale (Hamilton, 1960) and the 14‐item Hamilton Anxiety Rating Scale (Hamilton, 1959). Additional inclusion criteria were as follows: (a) No antipsychotic drugs and SSRIs were used; (b) 18 to 50 years old; (c) right‐handedness; (d) no history of neurological or other physical illness; (e) no history of other psychiatric disorders; (f) no history of psychoactive substances. Fifty‐six of the 85 OCD patients completed the 12‐week course of CBT.

We recruited 90 healthy volunteers using the Structured Clinical Interview from the DSM‐4. The control group and patient group were matched for age, sex, and handedness. None of the participants in the healthy controls group had any neurological and physical illnesses and psychiatric disorders. The clinical and demographic data are shown in Tables 1 and 2. This study was approved by the Ethics Committee of Beijing Anding Hospital and the second affiliated hospital of Xinxiang Medical University. Participants signed informed consent.

**Table 1:** Demographic and clinical characteristics of participants

Characteristic	OCD patients (*n* = 85)	Controls (*n* = 90)	*p*
Age (years)	27.6 ± 6.7 (19–48)	28.2 ± 6.8 (18–45)	0.645
Gender (M/W)	52/33	65/25	0.045
Education (years)	13.8 ± 1.2	13.9 ± 1.1	0.564
Brain size (mm^3^)	1.15 ± 0.15 x 107	1.18 ± 0.17 × 107	0.372
Illness duration (month)	70.06 ± 63.67	NA	
Total Y‐BOCS score	24.19 ± 6.32	NA	
Y‐BOCS1 score	13.66 ± 3.21	NA	
Y‐BOCS2 score	10.53 ± 5.64	NA	
HAMD score	7.28 ± 3.59	NA	
HAMA score	8.12 ± 3.34	NA	

Note

HARS, Hamilton Anxiety Rating Scale; HDRS, Hamilton Depression Rating Scale; Y‐BOCS, Yale‐Brown Obsessive Compulsive Scale.

**Table 2:** Clinical characteristics of pretreatment and posttreatment OCD patients

	Pretreatment（*n* = 56）	Posttreatment	*t*	*p*
Total Y‐BOCS score	23.62 ± 5.73	11.36 ± 5.76	14.48	0.000
Y‐BOCS1 score	13.05 ± 3.10	6.33 ± 3.32	13.03	0.000
Y‐BOCS2 score	10.56 ± 5.06	5.03 ± 3.44	10.08	0.000
HAMD score	6.41 ± 3.45	2.41 ± 2.79	8.09	0.000
HAMA score	7.49 ± 3.63	2.74 ± 2.74	9.80	0.000

### 2.2 Treatment with CBT

Patients were randomly allocated to a therapist and received a 12‐week individual CBT program that consisted of 14 sessions. None of the patients took psychoactive medication during the treatment period. CBT was provided by four experienced therapists at postgraduate level who had completed 320 hr of CBT training. Prior to the study, all therapists received one month of training that was facilitated by qualified CBT trainers. The therapists provided the treatment in line with the guidance provided in the treatment manual of CBT for OCD and accepted 1 hr of supervision each week with a senior therapist for the duration of the research period. This study compiled a CBT manual based on the researchers’ previous CBT trainings and the existing treatment manual of CBT for OCD (Whittal et al., 2005; Wilson & Chambless, 2005). This treatment manual consisted of 14 sessions, and each session was 60 min in duration (Yang et al., 2015). The CBT program was deemed effective when there was a decrease of more than 35% in the total Y‐BOCS score, in accordance with practice guidelines for the treatment of OCD (Koran, Hollander, Nestadt, & Simpson, 2007).

### 2.3 Measurements and evaluation

Four raters independently assessed patients using the Y‐BOCS, HAMD, and HAMA scores at baseline and at 12 weeks. The raters were not involved in the CBT interventions. Prior to the study, the four raters participated in joint training sessions and had an interrater reliability (i.e., intraclass correlation) of >0.85 for all assessment scales with respect to ten OCD patients.

### 2.4 Brain image acquisition

All patients and healthy controls were scanned for DTI data using a SIEMENS 3‐T scanner at the State Key Laboratory of Cognitive Neuroscience and Learning of Beijing Normal University or the second affiliated hospital of Xinxiang Medical University. Fifty‐six patients were scanned before and after the 12‐week course of CBT. Diffusion‐weighted images were acquired using whole‐brain echo‐planar imaging (EPI) sequence. The parameters were as follows: TR = 7,200 ms; TE = 104 ms; NEX = 8; in‐plane acquisition matrix = 128 × 128; field of view (FOV) = 230 × 230 mm^2^; 2.5 mm slice thickness with no interslice gap; 49 axial slices. The diffusion sensitizing gradients were applied along 64 noncollinear directions (b = 1,000 s/mm^2^), together with an acquisition without diffusion weighting (b = 0 s/mm^2^).

### 2.5 Brain imaging data processing

We adopted a voxel‐based analysis (VBM) in our study，which had been used in previous studies (Menzies et al., 2008; Szeszko et al., 2005; Yoo et al., 2007). The data preprocessing steps were as follows: All the data were processed by using Statistical Parametric Mapping (SPM8; http://www.fil.ion.ucl.ac.uk/spm/) and software tools from the Functional MRI of the Brain (FMRIB) software library FSL (FSL, http://www.fmrib.ox.ac.uk/fsl/). (a) The diffusion data set was prealigned to correct for head motion and the effects of gradient coil eddy currents using the eddy correct toolbox in FSL (Liao et al., 2010). (b) The diffusion tensor at each voxel was calculated by using the FMRIB diffusion toolbox in FSL. The resulting FA images were transformed into Montreal Neurological Institute (MNI) standard space with SPM8 by means of the following steps: the b = 0 images were coregistered with the T1 image for that individual, the same coregistration parameters were applied to the FA maps (in the same space as the b = 0 images), each individual's T1 image was then normalized to the SPM T1 template in MNI standard space, and the same normalization parameters were then applied to the coregistered FA images. (c) FA images were smoothened with a 6 mm full width at half maximum Gaussian kernel in order to decrease spatial noise and compensate for the inexact nature of normalization. (d) All the images were resampled with a voxel size of 2 × 2×2 mm^3^. In order to use in the two‐sample *t* test, a binary WM mask was made by calculating a mean FA image with a threshold FA value of 0.25 from FA images of all the participants. (e) We performed a whole‐brain, voxel‐based analysis of diffusion tensor MRI using the normalized and smoothed FA maps obtained using Statistical Parametric Mapping.

### 2.6 Statistical analysis

An independent *t* test was performed with the age, gender, highest diploma level, and brain size of each participant as covariates to explore differences in FA values between OCD patients and HCs. A false discovery rate (FDR) procedure was performed to correct for multiple comparisons at a q value of 0.05. The pre and posttreatment differences in FA values were compared for patients who responded to CBT using a paired *t* test. We further examined the relationship between FA values and the Y‐BOCS scores for OCD groups using Pearson's correlation analysis with age, gender, highest diploma level, and brain size as covariates.

## 3 RESULTS

### 3.1 CBT treatment outcome

Patients showed significant improvement posttreatment compared with pretreatment. In total, 38 of the 56 patients were identified as having responded to this change (i.e., defined as a minimum reduction of 35% in the Y‐BOCS score).

### 3.2 FA analysis in pretreatment OCD patients and HCs

**Figure 1:**
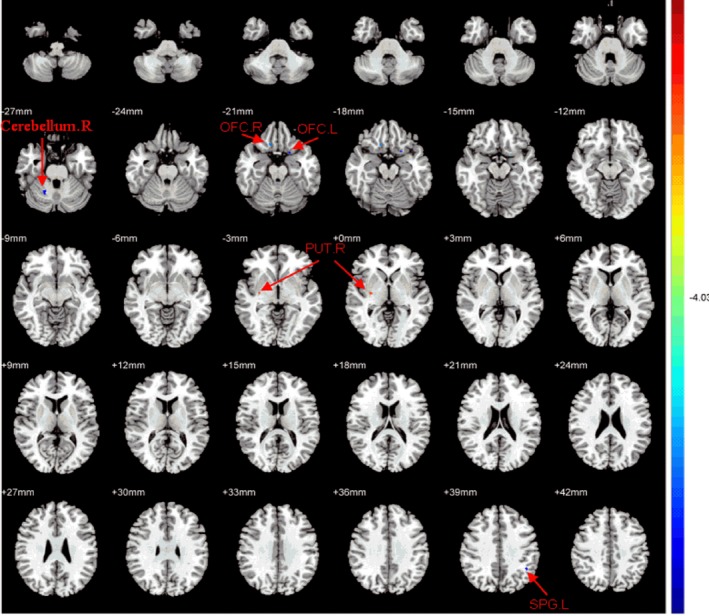
Regions with significant differences in FA value between OCD patients and NCs. The OCD patients showed decreased FA values in the right orbital frontal cortex, left orbital frontal cortex, right cerebellum, left superior parietal gyrus, higher FA values in the right putamen nucleus white matter. T‐score bars were shown on the right. Red and blue denote higher and lower FA values, respectively (*p* < 0.001, corrected)

Compared with HCs, OCD patients showed significantly decreased FA values in the right orbital frontal cortex, left orbital frontal cortex, right cerebellum, and left superior parietal gyrus, while higher FA values were exhibited in right putamen nucleus WM (Table 3, Figure 1).

**Table 3:** Comparisons of the FA value among the OCD and control groups

Number	Region	MNI coordination (mm)			*t*	*p*
		*X*	*Y*	*Z*		
1	Orbital frontal cortex (R)	12	22	−20	−5.45	<0.001
2	Orbital frontal cortex (L)	−18	11	−20	−7.69	<0.001
3	Cerebellum.R	20	−53	−28	−7.03	<0.001
4	Superior parietal gyrus (L)	−47	−52	39	−6.77	<0.001
5	Putamen nucleus (R)	32	−10	−1	4.34	<0.001

### 3.3 FA analysis in pretreatment and posttreatment OCD patients

**Figure 2:**
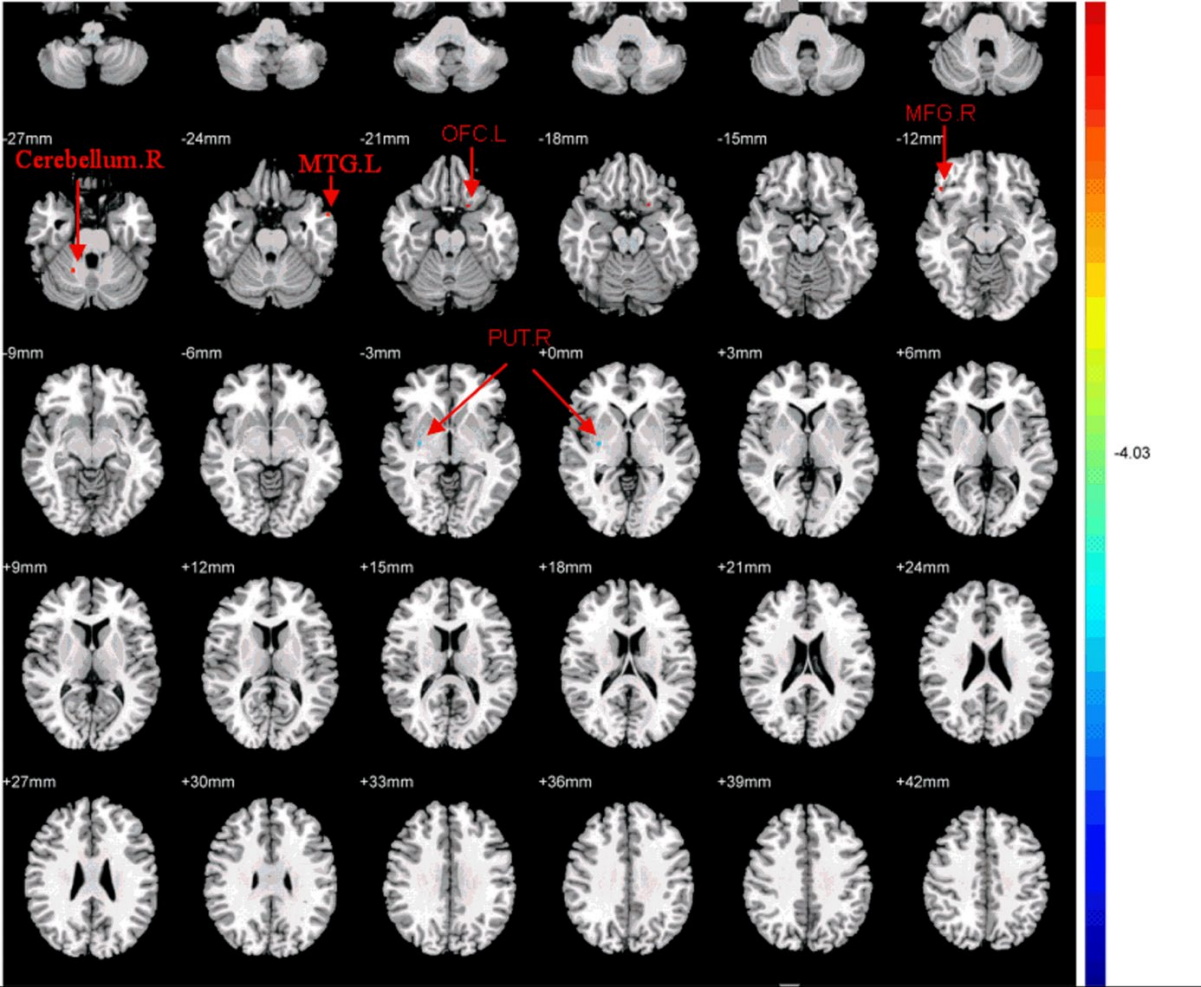
Regions with significant differences in FA value between pre and posttreatment OCD patients (*p* < 0.001, corrected). The OCD patients showed significantly higher FA values in the right middle frontal gyrus, left orbital frontal cortex, right cerebellum, left middle temporal gyrus and decreased FA values in the right putamen nucleus after CBT. T‐score bars were shown on the right. Red and blue denote higher and lower FA value, respectively

This study investigated alterations in FA values in patients who responded to CBT. In comparison with pretreatment, posttreatment OCD patients showed significantly higher FA values in the right middle frontal gyrus, left orbital frontal cortex, right cerebellum, and left middle temporal gyrus, and decreased FA values in right putamen nucleus WM (paired *t* test, *p < *0.05, corrected; Table 4, Figure 2). Among these brain areas, OCD patients exhibited changes in WM microstructure in the left orbital frontal cortex, right cerebellum, and right putamen nucleus WM. Furthermore, we compared the posttreatment FA values of these three brain regions for OCD patients and HCs using an independent *t* test. The findings revealed no difference in FA values for these regions.

**Table 4:** Comparisons of the FA value among the pre and posttreatment OCD patients

Number	Region	MNI coordination (mm)			*t*	*p*
		*X*	*Y*	*Z*		
1	Middle frontal gyrus (R)	48	30	−12	5.16	<0.001
2	Orbital frontal cortex (L)	−18	11	−20	5.47	<0.001
3	Cerebellum (R)	20	−53	−28	4.60	<0.001
4	Middle temporal gyrus (L)	−57	6	−24	4.68	<0.001
5	Putamen nucleus (R)	32	−10	−1	−5.36	<0.001

### 3.4 Correlation between FA values and Y‐BOCS scores, and the efficacy of CBT for OCD

#### 3.4.1 Correlation between abnormal FA values and Y‐BOCS scores at baseline

**Figure 3:**
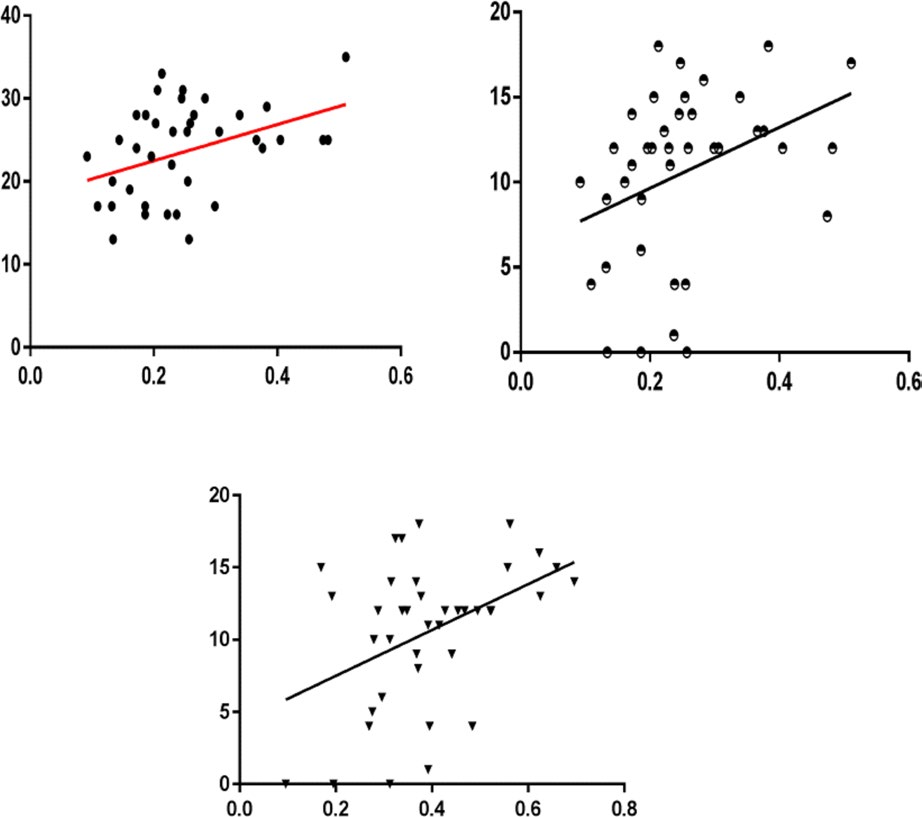
The correlation between the FA values and Y‐BOCS scores at baseline in OCD patients. Plots showing the FA values were significantly positively correlated with Y‐BOCS

For the OCD group, we carried out Pearson's correlation analysis with a significance level set at *p* < 0.05 in order to examine the correlation between FA values that show significant group differences and the Y‐BOCS scores. We found that FA values in left orbital frontal cortex WM were significantly positively correlated with Y‐BOCS (*r = *0.39, *p* = 0.014) and Y‐BOCS compulsions subscales (*r = *0.362, *p* = 0.023) and FA values in right putamen nucleus WM were significantly positively correlated with Y‐BOCS compulsions subscales (*r = *0.427, *p* = 0.007; Figure 3).

#### 3.4.2 Correlation between abnormal FA values and efficacy of CBT

We also examined the correlation between FA values, changes in FA values, and the efficacy of CBT using Pearson's correlation analysis and the significance level was set at *p < *0.05. We found that FA values in brain cortex WM at baseline were not significantly correlated with the percentage reduction in the Y‐BOCS score after CBT. Furthermore, the percentage change in FA values in the left middle temporal gyrus was significantly positively correlated with the percentage reduction in Y‐BOCS compulsions subscales (*r* = 0.418, *p = *0.021), while the percentage change in FA values in the left orbital frontal cortex and right putamen nucleus WM was significantly negatively correlated with the percentage reduction in Y‐BOCS obsessions subscales(*r* = −0.373, *p = *0.019) and the percentage reduction in Y‐BOCS compulsions subscales (Compulsions subscale; *r* = −0.409, *p* = 0.01), respectively. In addition, no significant correlation was found between the change in depression and anxiety symptoms, the baseline FA values, and changes in FA values in those regions.

## 4 DISCUSSION

This is the first DTI study to investigate the effects of CBT on WM in unmedicated OCD patients. At baseline, we found that patients with OCD showed WM abnormalities located within the fronto‐striato‐thalamo‐cortical circuit (i.e., orbital frontal cortex, putamen nucleus) and other brain regions (i.e., cerebellum, superior parietal gyrus). Following an effective program of CBT for OCD patients, the recovery of WM fiber connections occurred in some brain regions and was correlated with an improvement in obsessive–compulsive symptoms.

### 4.1 Abnormalities of white matter microstructure in unmedicated patients with OCD

In our study, we found that OCD patients showed significantly decreased FA values in the WM in right orbital frontal cortex, left orbital frontal cortex, right cerebellum, and left superior parietal gyrus, while higher FA values were observed in WM in right putamen nucleus white matter compared with healthy controls. Current understanding acquired from functional and structural neuroimaging emphasizes abnormalities of the striato‐thalamo‐orbitofrontal circuits in the pathophysiology of OCD (Saxena, Bota, & Brody, 2001; Saxena et al., 1999). The OFC is a region of interest and plays an important role in the pathophysiological mechanism of OCD. Neuropsychological studies have indicated that the OFC plays an important role in response inhibition and behavioral suppression (Kashyap, Kumar, Kandavel, & Reddy, 2013). Many previous studies have highlighted abnormalities in the OFC with respect to functional activation (Mazziotta, Phelps, & Pahl, 1988), gray matter morphology (Choi et al., 2004; Szeszko et al., 1999), and functional alterations associated with the symptomatology of OCD (Nakao et al., 2005). Our results showed abnormalities of white matter microstructure in the OFC of OCD patients, and FA values in the WM in left OFC were significantly positively correlated with the Y‐BOCS score and Compulsions subscale score. Atmaca et al. (2011) examined the relationship between defense styles and OFC volumes and found a significant relation between the scores of immature defense mechanisms and OFC volumes in the patient group, particularly with respect to the left OFC. The findings suggested that the left OFC might be associated with treatment resistance in patients with OCD. It is possible that this abnormality may contribute to poor inhibitory control in patients with OCD, resulting in recurrent thoughts and repetitive behaviors. In addition, this abnormality can be used as an important biological indicator of OCD in order to predict the severity of obsessive–compulsive symptoms.

The putamen is a part of the lenticular nucleus in the striatum, which is mainly related to movement of the hands and feet. Although it is a component of the classical abnormal loop of OCD, few studies have examined the role of the putamen in OCD and little research has investigated microstructural WM of the putamen. Zarei et al. (2011) compared the volume of gray matter in 26 cases of OCD and 26 controls using the voxel‐based morphometry (VBM) method. Compared with the control group, the study found gray matter volume increases in the right putamen in patients with OCD. Using fMRI, Lázaro et al. (2008) found significantly higher brain activation in the putamen of OCD patients and demonstrated an improvement in symptoms following a 6‐month course of drug treatment. Moreover, activity of the lateral putamen was reduced. FMRI studies and a meta‐analysis also implicated the putamen during both symptom provocation and initiation of compulsive behaviors (Banca et al., 2015; Ilieva, Thorsen, & Michel, 2018). A meta‐analysis reported that the left putamen showed significantly less FA in OCD patients than healthy controls (Eng, Sim, & Chen, 2015). Our results also showed abnormalities of WM microstructure in the right putamen, thus further validating the abnormalities of the fronto‐striato‐thalamo‐cortical loop in OCD. Kwon et al. (2003) has found that the function of this loop is related to the symptoms. Our results showed that FA values in the WM in right putamen nucleus were significantly positively correlated with the Compulsions subscale scores. It is necessary to further confirm these results by utilizing a combination of methods such as neuroimaging, animal models studies, and neuropsychological tests to explore whether the damage is associated with compulsive behavioral symptoms such as repetitive hand washing, repetitive examination.

We found that the value of FA in right cerebellar WM was decreased in OCD patients. The cerebellum is located in the rear of the cerebral hemisphere, covering the pontine and the medulla, between the mesencephalon and the medulla. The cerebellum is involved in muscle tension and maintaining body posture, as well as in the coordination of random movements through its rich afferent and efferent fibers with the cerebrum, the brain stem, and the spinal cord. The cerebellum is also implicated in cognitive function, such as sports learning memory, language, executive functioning (Stoodley, 2012). There are few studies investigating the cerebellum in OCD patients. Kim and his colleagues (Kim et al., 2001) found decreased gray matter density in the cerebellum of OCD patients; Pujol et al. (2004) found increased gray matter volume in the cerebellum of OCD patients; Nabeyama et al. (2008) found that activity in the cerebellum was enhanced in the Stroop‐task state and the symptom‐activation state. However, while no study has examined microstructural WM changes in the cerebellum in OCD patients, studies investigating changes in cerebellar volume in patients have suggested that the structural basis of OCD may be explained by WM abnormalities. Our study was first verified using DTI technology. In combination with its function, we hypothesized that WM lesions (WMLs) in the texture of the cerebellum may cause dysfunction, resulting in recurrent compulsions and diminished inhibition.

Comparable to healthy controls, we also found a decrease in the FA value of WM in the left parietal superior gyrus in patients with OCD. The parietal cortex is known to involve attention and visual–spatial processes as well as various executive functions, such as task switching, planning, and working memory (Posner & Petersen, 1989). The DTI study examining the role of the parietal lobe in OCD yielded few findings. Utilizing the VBM method, Menzies et al. (2008) found that the FA value of WM fiber in the right parietal inferior gyrus of patients was lower than that of the control group; Szeszko et al. (2005) found that the FA value of WM in the bilateral parietal lobe was reduced, and the decreased FA value was associated with the severity of symptoms. Our results also demonstrated that there are microstructural WM abnormalities in OCD, which further verifies previous studies. While the role of the parietal lobe in the pathogenesis of OCD remains unclear, we posited that the parietal lobe is implicated in cognitive functioning including attention and cognitive transformation. According to a meta‐analysis, functional changes in the parietal lobe among patients with OCD are likely associated with cognitive impairment. As a result, our study suggested that abnormalities in WM microstructure in OCD patients are likely implicated in the pathophysiology of OCD. The brain's WM is the carrier for the transmission of information, and the connection between these nerve fibers in the brain provides the material basis for maintaining normal brain functioning. In the event that WM integrity is damaged or abnormal in the brain region, the delay or interruption of information transmission is likely to create an obstacle or result in the loss of a particular brain function. This research suggested that abnormalities in WM integrity in some brain regions may explain the structural basis of abnormal brain functioning in patients with obsessive–compulsive symptoms.

In conclusion, this study utilized the DTI method to investigate WM changes in patients with OCD and found that patients showed decreased FA of WM fiber in the bilateral OFC, right cerebellum, and left superior parietal gyrus and higher FA of WM fiber in the right putamen nucleus, suggesting that these brain regions are implicated in the pathophysiology of OCD.

### 4.2 Changes in white matter microstructure in OCD after CBT

At present, relatively few studies have examined WM integrity both before and after treatment with medication or CBT. Fan et al. (2012) found that drug treatment resulted in a reduction in radial diffusivity (RD) in the left striatum and right midbrain as well as a reduction in mean diffusivity (MD) of the right midbrain. However, an additional result showed that decreased FA values in WM of the nucleus accumbens may not be reversible by adequate treatment in OCD patients (Feng, 2014). The study conducted by Yoo et al. (2007) noted that WM alterations may be partly reversible with citalopram treatment. Our results showed that WM alterations in some regions (i.e., left orbital frontal cortex, right cerebellum, right putamen nucleus) can be partly reversible following an effective course of CBT. The percentage change in FA values of WM in the left orbital frontal cortex and right putamen nucleus was significantly negatively correlated, and the left middle temporal gyrus was significantly positively correlated with the percentage reduction in Y‐BOCS scores. The orbitofrontal cortex (OFC) is a region of interest and plays an important role in the pathophysiological mechanisms of OCD. A single‐photon emission computed tomography study found that baseline regional cerebral blood flow in the bilateral OFC was significantly correlated with a change in clinical symptoms after CBT (Yamanishi et al., 2009). Using functional MRI, Nakao et al. (2005) found that after a 12‐week treatment program of CBT, the activation intensity of the orbital frontal cortex, the dorsolateral prefrontal cortex, and the anterior cingulate gyrus caused by symptoms was weakened, while the activation intensity of the temporal cortex and cerebellum increased in OCD patients. In another study, the cerebral volume of 29 pediatric patients with OCD and 29 healthy controls was compared using VBM both before and after CBT (Huyser et al., 2013). In comparison with healthy controls, the results revealed an increase in orbitofrontal gray matter in OCD patients after CBT, and orbitofrontal gray matter volume was positively correlated with changes in symptom severity after CBT. The putamen is a part of the classical anomalous loop in OCD. Hoexter et al. (2012) found volume reductions in the left putamen in OCD patients and that the volume in the putamen increased after fluoxetine treatment. The temporal lobe is associated with speech integration, emotions, executive functioning, and memory (Nakao et al., 2009). It is connected with the orbitofrontal cortex, ventral prefrontal lobe, dorsolateral prefrontal lobe to form the interconnected fronto‐striato‐thalamo‐cortical loop, and changes within these functional connections may disrupt the balance between direct or indirect pathways, thus resulting in compulsive behavior (den Braber et al., 2011). In a functional MRI study, Sanematsu et al. (2010) found that after 12 weeks of fluvoxamine treatment, activity in the left temporal gyrus, left cuneate, right cerebellum, and right frontal cortex was enhanced during active symptoms of OCD, and activity in the left temporal gyrus and right cerebellum could predict the response of patients to fluvoxamine. In another study, Olatunji et al. (2014) found that the anterior temporal pole and amygdala showed the strongest association with a better treatment response with CBT. As outlined in these studies, pharmacological agents and psychotherapeutic interventions may lead to structural cerebral changes in patients with OCD, which was supported by our findings.

The neural mechanisms of CBT for the treatment of OCD are not yet clear. Previously, it has been shown that the serotonergic modulation of neurotransmission using selective serotonin reuptake inhibitors (SSRIs) can regulate neuroplasticity in a variety of cortical and subcortical regions implicated in OCD (Czéh et al., 2007; Soumier, Banasr, Goff, & Daszuta, 2009). The present study found that OCD patients exhibited changes in WM microstructure in the left orbital frontal cortex, right cerebellum, and right putamen nucleus WM after CBT. There is no precise understanding of direct correlation between these changes and the therapeutic effects of CBT. This study suggests that abnormalities in WM microstructure in these regions offer further support for the role of WM in the pathophysiology of OCD which may be partly reversed by CBT. The changes in WM can help us to explore the imaging mechanisms of CBT for the alleviation of obsessive–compulsive symptoms, while a much more robust methodology is required to investigate true pathophysiological mechanisms or mechanisms of change in therapy (Kazdin., 2007).

## 5 LIMITATIONS

Several limitations must be considered. First, patients with OCD underwent two MRI scans that included one pretreatment and one posttreatment scan, while healthy controls underwent only one scan. Second, VBM has excellent repeatability and can automatically analyze the DTI parameter map of the whole brain. However, this method has some limitations. For example, there is no standardization algorithm in SPM software for large anisotropic images, which may lead to false‐positive results and low accuracy in the registration of images into standardized space. In addition, smoothing in data processing may affect the results of the analysis, and multiple calibrations during processing will also affect the sensitivity of results. Third, the sample utilized in our study is comparatively small and the generalizability of the results needs further verification by using a larger sample size. We excluded individuals with a history of other comorbid psychiatric disorders including mood disorders. The exclusion of comorbid disorders including anxiety disorder, major depressive disorder, or dysthymia may have distorted the representativeness of the sample. For example, 60%–80% of people with OCD had a history of MDD. However, the inclusion and exclusion criteria were formulated so as to ensure homogeneity of participants and to control for the impact of comorbid disorders on the results. Fourth, while studies have outlined the clinical symptoms of different subtypes of OCD, we did not classify OCD patients were classified into subtypes according to their clinical symptoms, such as obsessive–compulsive disorder and compulsive testing, cleaning, hoarding.

## 6 CONCLUSION

This study utilized the DTI method to investigate WM abnormalities in patients with OCD both before and after CBT. We found that patients showed WM abnormalities in the fronto‐striato‐thalamo‐cortical circuit (i.e., orbitofrontal cortex, putamen nucleus) and other brain regions (i.e., cerebellum, superior parietal gyrus). OCD patients showed recovery of WM fiber connections in some regions (i.e., the left orbitofrontal cortex, right cerebellum, right putamen nucleus) after an effective program of CBT, and the recovery of WM fiber connections was correlated with an improvement in obsessive–compulsive symptoms. Our data suggest that such changes can help us to explore the imaging mechanisms of CBT for the alleviation of obsessive–compulsive symptoms.

## CONFLICT Of INTEREST

All authors declare no conflict of interest.

## AUTHOR CONTRIBUTIONS

Zhaoxi Zhong, Dai Zhang, LuXian Lv, and Zhanjiang Li contributed to the conception and design of the study and the acquisition, analysis, and interpretation of data. RuiXiang Cao and Ping Li contributed to the conception and design of the study and the acquisition of data. XiangYun Yang and Zhanjiang Li contributed to the conception and design of the study and the analysis of data. Zhaoxi Zhong and XiangYun Yang wrote the article, which all other authors reviewed. All authors gave approval for publication.

## CONSENT FOR PUBLICATION

All authors have read and approved this version of the article for publication.
